# Analysis of the Clinical Outcome of Microlumbar Decompression in Degenerative Grade-1 Spondylolisthesis versus Stable Lumbar Canal Stenosis

**DOI:** 10.1055/s-0045-1810036

**Published:** 2025-08-18

**Authors:** Ajaybir Singh

**Affiliations:** 1 Department of Orthopedics, Guru Gobind Singh Medical College and Hospital, Baba Farid University of Health Sciences, Faridkot, Punjab, India

**Keywords:** intervertebral disc degeneration, low back pain, sciatica, spinal diseases, spondylolisthesis, ciática, degeneração do disco intervertebral, doenças da coluna vertebral, dor lombar, espondilolistese

## Abstract

**Objective:**

To assess the clinical outcomes of a non-fusion decompression procedure in degenerative low-grade spondylolisthesis versus stable lumbar canal stenosis.

**Methods:**

The study analyzed 50 cases of lumbar degenerative pathology that underwent decompression involving a single level. Dynamic X-ray assessment was employed to evaluate instability. Group 1 included stable lumbar stenosis, while group 2 included Meyerding grade-1 degenerative spondylolisthesis. Two years postmicrolumbar decompression, the final functional outcomes were assessed using the Oswestry Disability Index (ODI) and the Visual Analog Scale (VAS) for backache and leg pain.

**Results:**

Group 1 included 25 cases with a mean ODI of 75.36 ± 13.59, mean VAS for backache of 5.92 ± 3.45, and mean VAS for leg pain of 8.92 ± 1.81. Group 2 included 25 cases with a mean ODI of 68.75 ± 11.81, mean VAS backache of 8 ± 1.22, and mean VAS leg pain of 7.87 ± 1.57. At 2 years, group 1's mean ODI improved to 22.64 ± 17.2 ( *p* < 0.0001), mean VAS backache reduced to 2.04 ± 1.86 ( *p* = 0.0002), and VAS leg pain reduced to 1.56 ± 1.97 ( *p* < 0.0001). Group 2 showed a mean ODI of 24 ± 10.6 ( *p* < 0.0001), backache mean VAS of 2.12 ± 1.43 ( *p* = 0.0009), and mean VAS leg pain of 2.56 ± 1.35 ( *p* = 0.0008). Both groups showed statistically comparable functional improvement.

**Conclusion:**

Microlumbar decompression yielded comparable functional outcomes in both groups. This procedure presents a viable option for preserving the integrity of lumbar motion segment in degenerative low grade (Meyerding grade 1) spondylolisthesis.

## Resumo

**Objetivo:**

Avaliar os desfechos clínicos de um procedimento de descompressão sem fusão em pacientes com espondilolistese degenerativa de baixo grau ou estenose estável do canal lombar.

**Métodos:**

O estudo analisou 50 casos de doença degenerativa lombar submetidos à descompressão de um único nível. A avaliação radiográfica dinâmica foi utilizada para determinação de instabilidade. O grupo 1 incluiu pacientes com estenose lombar estável e o grupo 2 incluiu pacientes com espondilolistese degenerativa de grau 1 de Meyerding. Dois anos após a microdescompressão lombar, os desfechos funcionais foram analisados segundo o Índice de Incapacidade de Oswestry (ODI, do inglês Oswestry Disability Index) e a Escala Visual Analógica (EVA) de dor lombar e nos membros inferiores.

**Resultados:**

O grupo 1 incluiu 25 casos com ODI médio de 75,36 ± 13,59 e EVA média de dor lombar de 5,92 ± 3,45 e de dor nos membros inferiores de 8,92 ± 1,81. O grupo 2 incluiu 25 casos com ODI médio de 68,75 ± 11,81 e EVA média de dor lombar de 8 ± 1,22 e de dor nos membros inferiores de 7,87 ± 1,57. Em 2 anos, o ODI médio do grupo 1 melhorou para 22,64 ± 17,2 ( *p* < 0,0001) e a EVA média de dor lombar caiu para 2,04 ± 1,86 ( *p* = 0,0002) e de dor nos membros inferiores diminuiu para 1,56 ± 1,97 ( *p* < 0,0001). O grupo 2 apresentou ODI médio de 24 ± 10,6 ( *p* < 0,0001) e EVA média de dor lombar de 2,12 ± 1,43 ( *p* = 0,0009) e de dor nos membros inferiores de 2,56 ± 1,35 ( *p* = 0,0008). Ambos os grupos apresentaram melhora funcional estatisticamente comparável.

**Conclusão:**

A microdescompressão lombar produziu desfechos funcionais comparáveis em ambos os grupos. Este procedimento é uma opção viável para preservação da integridade do segmento de movimento lombar na espondilolistese degenerativa de baixo grau (grau 1 de Meyerding).

## Introduction

Segmental instability of the lumbar spine, a consequence of degenerative conditions, is a well-recognized issue. It frequently affects the L4–5 and L5–S1 levels, which possess the highest range of motion. Patients exhibiting radiographically unstable degenerative spines typically receive a recommendation for surgical stabilization as a treatment option. 1 However, the addition of fusion brings its own set of complications. Therefore, a non-fusion option for decompression is desirable in degenerative lumbar spine when indicated. 2

X-rays, computed tomography (CT), and magnetic resonance imaging (MRI) are commonly employed diagnostic tools to assess degenerative lumbar pathologies. While these radiological investigations are inherently static, dynamic X-rays are increasingly utilized to detect radiological instability. Additionally, the lumbar spine exhibits a wide range of normal motion, which may inadvertently guide treatment toward fusion surgery. This predicament is particularly pertinent in instances of low-grade (Meyerding grade 1) degenerative spondylolisthesis. Various studies have attempted to define translational, rotational, or angular abnormalities on X-rays. 3 4 5 A micro lumbar decompression and discectomy procedure is designed to minimize bone loss, preserve motion segments, and provide adequate decompression of neural elements. 6

## Materials and Methods

The current work was approved by institutional Ethics Committee under registration nr. ECR/836/lnst./PB/2016/RR-20, nr. IEC/29.

Fifty patients who underwent lumbar spine surgery for a degenerative lumbar spine pathology at a single level were included. Magnetic resonance imaging was employed to evaluate the extent of pathology. Additionally, preoperative flexion and extension X-rays of the lumbar spine were conducted to assess any sign of radiological instability. Lateral flexion-extension radiographs were taken as described by Putto and Tallroth. 4

To assess the stability of the motion segment, translational and angular motion were calculated on radiographs according to Dupuis et al. 5 Postoperative clinical follow-up was conducted at 6 weeks, 3 and 6 months, and 1 and 2 years.

Clinical evaluation pre and postoperatively was performed utilizing the Visual Analog Scale (VAS) to assess leg pain and backache separately, and functional outcome assessment was performed by the Oswestry Disability Index (ODI).

The included cases were adult patients (aged 18 and above) who underwent single-level spine surgery for degenerative lumbar canal stenosis (LCS), prolapsed intervertebral disc (PIVD), and degenerative low-grade spondylolisthesis (Meyerding grade 1).

The exclusion criteria were defined as cases of spinal instability presenting with vertebral fractures, spinal tumors, degenerative spondylolisthesis Meyerding grade 2 or more, spinal infections, lytic listhesis, pars fractures, cases requiring revision lumbar spine surgery, associated cervical or thoracic segment involvement requiring surgical intervention, and intradural pathologies. Ethical committee approval was obtained for conducting this research study.

### Surgical Method

Microlumbar disectomy was performed with the patient in prone position under general anesthesia. The level for decompression was marked using fluoroscopy. A midline approach to the posterior lumbar spine was used. After subperiosteal dissection, the microscopically-assisted laminotomy was performed using a burr and a Kerrison roungeur. The hypertrophic ligamentum flavum was removed for central canal decompression. For lateral recess decompression, a partial facet undercutting was added when required. In all patients, the nerve root was visualized and adequately decompressed. The discectomy procedure was done after annulotomy. Closure was done in layers.

Postoperatively, the patient was mobilized on the day following surgery. Core strengthening exercises were initiated as tolerated by the patient. Physiotherapy and rehabilitation were gradually intensified in accordance with the patient's clinical improvement.

## Results

### Patient Demographics and Baseline Characteristics

A total of 53 patients participated in the study, but 3 patients were lost to follow-up. Group 1 consisted of 25 cases with radiologically stable lumbar segment, while group 2 included 25 cases diagnosed with degenerative low-grade spondylolisthesis (Meyerding grade 1), as assessed through dynamic X-rays. All patients underwent single-level midline microlumbar decompression. Clinical evaluations were conducted at 3 and 6 months, and 1 and 2 years postsurgery. The clinical outcomes of both groups were measured using the Visual Analog Scale (VAS) and the Oswestry Disability Index (ODI) at 2 years postsurgery.

### Age and Gender Distribution

The age distribution for both groups is presented in Table 1 . Both groups 1 and 2 had 25 patients each, with a comparable age distribution ( *p* = 0.6808 and *p* > 0.05, respectively, as per the Fisher's exact test). Table 2 summarizes the gender distribution: Group 1 included 12 males and 13 females, whereas group 2 comprised 14 males and 11 females. Statistical analysis demonstrated no significant difference in gender distribution between the 2 groups ( *p* = 0.6911, Fisher's exact test).

**Table 1:** Distribution of patients by age in groups 1 and 2

Age in years	Number (%)	
	Group 1	Group 2
≤ 30	3	2
31–40	4	7
41–50	10	2
51–60	6	10
≥ 61	2	4
Total	25	25

**Note** : Value of *p* : 0.6808 according to the Fisher's exact test, with an age cut-off of 50 years.

**Table 2:** Distribution of patients by gender in groups 1 and 2

Gender	Number (%)	
	Group 1	Group 2
Male	12	14
Female	13	11
Total	25	25

**Note** : Value of *p* : 0.6911 according to the Fisher's exact test.

### Lumbar Segment Involvement

Table 3 outlines the distribution of lumbar segment involvement. In group 1, the majority of cases involved the L4-to-L5 segment (14 cases), followed by L5 to S1 (8 cases), L3 to L4 (2 cases), and L2 to L3 (1 case). Group 2 exhibited involvement primarily in L4 to L5 (17 cases) and L5 to S1 (6 cases), with single cases at L2 to L3 and L3 to L4.

**Table 3:** Distribution of patients by level of involvement in groups 1 and 2

Diagnosis	Number	
	Group 1	Group 2
L2–L3	1	1
L3–L4	2	1
L4–L5	14	17
L5–S1	8	6

### Clinical Presentation

Patient's chief complaints are presented in Table 4 . In group 1, all 25 patients reported radiculopathy, with 19 experiencing additional backache along with radiating leg pain. Similarly, all 25 patients in group 2 reported radiculopathy, with 21 patients experiencing associated axial backache. Claudication distance was also recorded, revealing that 33 patients experienced claudication at ≤ 50 m, 7 patients at 100 m, and 10 patients at 200 m.

**Table 4:** Distribution of chief complaints in groups 1 and 2

Complaints	Number (%)	
	Group 1 N = 25	Group 2 N = 25
Backache	19 (76%)	21 (84%)
Radiculopathy	25 (100%)	25 (100%)

### Clinical Outcomes

Table 5 presents the clinical outcomes for group 1. The mean ODI score improved significantly from 75.36 ± 13.59 preoperatively to 22.64 ± 17.2 at 2 years postsurgery ( *p* < 0.0001). Visual analogue scale scores for backache and leg pain also demonstrated significant improvements. The mean VAS for backache reduced from 5.92 ± 3.45 preoperatively to 2.04 ± 1.86 ( *p* = 0.0002), while the mean VAS for leg pain decreased from 8.9 to 1.56 ( *p* < 0.0001).

**Table 5:** Comparison of ODI, VAS for backache and for leg pain preoperative and at the 2 years follow-up in group 1

Group	Group 1								*p* -value*
	Preoperative				Postoperative (at 2 years)				
Statistical measures	N	Mean	SD	Range	N	Mean	SD	Range	
ODI	25	75.36	13.59	50–96	25	22.64	17.2	0–54	< 0.0001
VAS for backache	25	5.92	3.45	0–10	25	2.04	1.86	0–5	0.0002
VAS for leg pain	25	8.92	1.61	2.50–10	25	1.56	1.97	0–5	< 0.0001

**Abbreviations:** ODI, Oswestry Disability Index; SD, standard deviation VAS, Visual Analogue Scale.

**Note** : *Wilcoxon signed rank test.

Clinical outcomes for group 2 are summarized in Table 6 . The mean ODI improved from 68.75 ± 11.81 preoperatively to 24 ± 10.6 at 2 years postsurgery ( *p* < 0.0001). The mean VAS for backache significantly decreased from 8 ± 1.22 to 2.12 ± 1.43 ( *p* = 0.0009). Furthermore, the mean VAS for leg pain improved from 7.87 preoperatively to 2.56 at 2 years postsurgery ( *p* = 0.0008).

**Table 6:** Comparison of ODI, VAS for backache and for leg pain preoperatively and after the 2-year follow-up in group 2

Group	Group 2								*p* -value
	Preoperative				Postoperative (at 2 years)				
Statistical parameters	N	Mean	SD	Range	N	Mean	SD	Range	
ODI	25	68.75	11.81	46–84	25	24	10.6	10–38	< 0.0001
VAS for backache	25	8	1.22	6–10	25	2.12	1.43	0–4	0.0009
VAS for leg pain	25	7.87	1.57	5–9	25	2.56	1.35	0–5	0.0008

**Abbreviations:** ODI, Oswestry Disability Index; SD, standard deviation VAS, Visual Analogue Scale.

**Note** : *Using Wilcoxon signed rank test.

Table 7 displays the comparative outcomes between the 2 groups at the 2-year follow-up. The mean ODI for group 1 (22.64 ± 17.2) was statistically comparable to group 2 (24 ± 10.6), with a *p* -value of 0.7924. Similarly, VAS scores for backache (2.04 ± 1.86 in group 1 versus 2.12 ± 1.43 in group 2; *p* = 0.7154) and leg pain (1.56 in group 1 versus 2.56 in group 2; *p* = 0.0945) showed no significant differences.

**Table 7:** Comparison of ODI, VAS backache and for leg pain between groups 1 and 2 at 2-year follow-up

Condition →	At the 2-year follow up								*p* -value*
Group →	Group 1				Group 2				
Statistical parameters →	N	Mean	SD	Range	N	Mean	SD	Range	
ODI	25	22.64	17.2	0–54	25	24	10.6	10–38	0.7924
VAS for backache	25	2.04	1.86	0–5	25	2.12	1.43	0–4	0.7154
VAS for leg pain	25	1.56	1.97	0–5	25	2.56	1.35	0–5	0.0945

**Abbreviations:** ODI, Oswestry Disability Index; SD, standard deviation VAS, Visual Analogue Scale.

**Note** : * Wilcoxon rank sum test.

### Follow-Up

At the final follow-up, 34 patients reported no claudication symptoms. Sixteen patients experienced minimal symptoms, such as heaviness or slight numbness in their legs, yet were able to walk distances exceeding one kilometer with conservative management of symptoms.

## Discussion

The degenerative instability process unfolds through three distinct phases: dysfunction, instability, and re-stabilization. Segmental instability arises from the degeneration of facets and discs, leading to laxity and resulting in abnormal motion under the physiological load of daily activities. 7 Panjabi 8 proposed a tri-modal concept of spinal stability and clinical manifestations of segmental instability are characterized by nonspecific symptoms, including low back pain accompanied by radicular pain, particularly during changes in posture.

A recent study by Van Grafhorst et al., 9 in 2025, reported the outcome of a 9-year follow-up of decompression without fusion in patients with lumbar spinal stenosis, with or without coexisting low-grade (grade I) degenerative spondylolisthesis. A total of 250 cases with low-grade spondylolisthesis had a 69% satisfaction rate, which was comparable to the group of 200 cases of degenerative stenosis (68%). Decompression alone yielded durable and satisfactory outcomes with reoperation rates as low as 7% in the spondylolisthesis group and 6% in the stenosis group.

A systematic review and meta-analysis of 6 randomized controlled trials (RCTs) was conducted by Abdel-Fattah et al. 10 in 2023 for the total of 531 patients with mean age of 66.2 years and mean follow-up of 27.4 months. The study showed decompression alone (DA) is not inferior to decompression with fusion (DF) in terms of pain, disability, or reoperation rates in elderly patients with spinal stenosis and low-grade degenerative spondylolisthesis. Decompression alone is associated with fewer surgical complications, shorter surgery, and lower perioperative burden. Hence, clinical decisions should balance surgical risks, patient comorbidities, and functional goals.

A finite element model study to assess the biomechanical impact of decompressions on lumbar stability was conducted by Liu et al. 6 The study tested normal lumbar segment versus grade 1 spondylolisthesis each with three surgical scenarios: hemilaminectomy, total laminectomy, one-third facetectomy. Hemilaminectomy and ⅓ facetectomy were biomechanically safe for low-grade spondylolisthesis, maintaining segment stability with minimal changes to spinal mechanics. However, total laminectomy significantly altered biomechanics and should be used cautiously in spondylolisthesis due to increased motion, disc pressure, and stress on posterior elements. Finite element modeling supported minimally invasive strategies that preserve stability while achieving decompression.

A multicenter RCT called Swedish Spinal Stenosis Study, published in 2024 by Karlsson et al., 11 presented long-term (5-year) outcomes of decompression alone (DA) versus decompression with fusion (DF) in patients with lumbar spinal stenosis with or without degenerative spondylolisthesis. Of 247 patients aged between 50 to 80 years, 124 in were included in DA group and 123 in DF group. The ODI, VAS, and European Quality of Life-5 Dimensions (EQ-5D) quality of life (QoL) assessment scores showed fusion does not provide superior clinical outcomes as QoL and leg pain relief were better with DA at 5 years. Decompression alone is preferable, offering similar or better results with lower complication risks, shorter hospital stays and fewer adjacent segment issues. Reoperations rates were similar in either group but different in cause, in DF group 24% (mainly adjacent level stenosis) and DA group 22% mainly due to restenosis.

The study by Kgomotso et al. 12 in 2024 aimed to evaluate the long-term effectiveness of decompression alone versus decompression with instrumented fusion for patients with degenerative lumbar spondylolisthesis (DS) over a 5-year follow-up. It was structured as a multicenter, non-inferiority RCT (NORDSTEN-DS) to determine if decompression alone is non-inferior to decompression with fusion. The study included 267 patients aged 18 to 80 years with lumbar spinal stenosis and spondylolisthesis of at least 3 mm at the stenotic level. Cases were randomly assigned to decompression alone ( *n* = 134) or decompression with fusion ( *n* = 133). The study concluded that decompression alone is non-inferior to decompression with instrumented fusion for treating degenerative lumbar spondylolisthesis at 5 years, without significant differences in clinical outcomes or reoperation rates.

A recent study by Unterfrauner et al. 13 aimed to compare the effectiveness of decompression alone versus decompression with fusion for patients with lumbar degenerative spondylolisthesis (DS) and spinal stenosis over a 3-year follow-up period, using data from the Lumbar Stenosis Outcome Study (LSOS). It employed a target trial emulation with benchmarking against the NORDSTEN-DS RCT to improve causal inference in observational data. The study concludes that decompression alone should be considered the primary surgical option for patients with lumbar DS and spinal stenosis, given that fusion did not provide additional benefits in terms of health-related QoL (HRQoL), pain reduction, or satisfaction at 3 years, while increasing physical therapy needs. This finding challenges the routine use of fusion in these cases, suggesting that decompression alone is effective and less resource intensive.

Systematic review and meta-analysis including 3 RCTs and 9 cohort studies by Cheng et al. 14 (2024) aimed to evaluate the efficacy and safety of decompression alone versus decompression plus fusion in patients with single-level lumbar spinal stenosis with spondylolisthesis. The analysis included 6,182 patients with 2,339 in the decompression-alone group and 3,783 in the decompression plus fusion group. The findings indicate that decompression alone is not inferior to decompression plus fusion for single-level lumbar spinal stenosis with spondylolisthesis. Given the shorter operation time, less intraoperative blood loss, and no increase in complications or reoperation rates, decompression alone is advocated as the primary surgical option.

A retrospective cohort study by Van Grafhorst et al. 2 aimed to assess the effectiveness of decompression alone as a treatment for symptomatic lumbar stenosis with low-grade degenerative spondylolisthesis and to evaluate the incidence and outcomes of reoperation, with or without subsequent instrumented fusion. A total of 934 patients undergoing surgery for lumbar spinal stenosis, including 253 patients with degenerative spondylolisthesis. All patients initially received decompression alone; only 3 patients with spondylolisthesis underwent primary decompression with fusion. After this first procedure, 80% of stenosis patients and 74% of spondylolisthesis patients reported satisfaction after initial decompression ( *p* = 0 *.* 059). Reoperation rates were 12% in the stenosis group (1.2% underwent fusion) and 17% in the spondylolisthesis group (38.1% underwent fusion). The study concludes that decompression alone is an effective treatment for symptomatic lumbar stenosis with low-grade degenerative spondylolisthesis. When reoperations were necessary, secondary decompression sometimes with extended resection of the superior arch was often sufficient. Fusion was only occasionally required, supporting the argument that instrumented fusion is not routinely necessary for most patients.

The study by Khashan et al. 15 aimed to evaluate the clinical outcomes, complication rates, and reoperation rates of minimally-invasive (MI) tubular decompression for lumbar spinal stenosis (LSS) with or without stable low-grade degenerative spondylolisthesis. It specifically examined if the presence of stable spondylolisthesis compromised the outcomes of MI decompression. Of 96 patients, 53 patients with stable low-grade degenerative spondylolisthesis and 43 patients with spinal stenosis. The presence of stable spondylolisthesis did not compromise clinical outcomes, increase complication rates, or elevate reoperation rates. The study concluded that MI tubular decompression is an effective and safe procedure for patients with lumbar spinal stenosis, regardless of the presence of stable low-grade degenerative spondylolisthesis.

Ghiselli et al. 16 conducted a study involving 215 cases of posterior lumbar fusion and observed a prevalence of symptomatic adjacent segment degeneration that necessitated either decompression or arthrodesis. The study predicted a 16.5% incidence of symptomatic adjacent segment degeneration at 5 years and a 36.1% incidence at 10 years. Carreon et al. 17 observed that decompression with fusion in individuals over the age of 65 years was associated with an elevated incidence of major complications, such as surgical site infections (prevalence, 10%) and minor complications, including urinary tract infections (prevalence, 34%). Furthermore, as the fusion levels increased, there was a concurrent rise in blood loss and operative time.

Our findings align with the conclusions of these studies, which all questioned the significance of fusion and advocated for a favorable perspective on a non-fusion and a less invasive decompression approach.

However, our study has some limitations, such as its retrospective nature, which restricts the generalizability of its findings. While dynamic flexion-extension X-rays of the lumbar spine are commonly employed in decision-making processes for lumbar fusion, radiographic parameters such as global sagittal balance evaluation are increasingly utilized. Absence of a fusion group is another limitation, as results depicted are only for a non-fusion surgery.

## Conclusion

Lumbar spine bears the brunt of body weight as compared with other spinal regions, while simultaneously facilitating a wide range of flexion and extension movements. Consequently, preserving lumbar motion is important. Surgical procedures aimed at preserving the motion of the lumbar spine have garnered significant attention, encompassing a range of techniques. It is crucial to identify a patient population that may benefit from avoiding a fusion procedure. In our study, a comparative analysis of degenerative low grade spondylolisthesis versus a stable lumbar canal stenosis undergoing a similar non fusion surgical procedure demonstrated comparable clinical outcomes. Consequently, microlumbar decompression emerges as a viable treatment option for preserving the lumbar motion segment in degenerative low-grade spondylolisthesis.

## References

[1] Frymoyer J W Selby D K Segmental instability. Rationale for treatment Spine 1985 10 03 280 286 10.1097/00007632-198504000-00017 3992349

[2] Van Grafhorst J MP Dijkerman M L Peul W C Vleggeert-Lankamp C LA Symptomatic lumbar stenosis due to low-grade degenerative spondylolisthesis can effectively be treated with mere decompression Acta Neurochir (Wien) 2023 165 08 2145 2151 10.1007/s00701-023-05667-7 37410183 PMC10409655

[3] Pitkänen M T Manninen H I Lindgren K A Sihvonen T A Airaksinen O Soimakallio S Segmental lumbar spine instability at flexion-extension radiography can be predicted by conventional radiography Clin Radiol 2002 57 07 632 639 10.1053/crad.2001.0899 12096864

[4] Putto E Tallroth K Extension-flexion radiographs for motion studies of the lumbar spine. A comparison of two methods Spine (Phila Pa 1976) 1990 15 02 107 110 10.1097/00007632-199002000-00011 2139240

[5] Dupuis P R Yong-Hing K Cassidy J D Kirkaldy-Willis W H Radiologic diagnosis of degenerative lumbar spinal instability Spine (Phila Pa 1976) 1985 10 03 262 276 10.1097/00007632-198504000-00015 3992347

[6] Liu R He T Wu X Tan W Yan Z Deng Y Biomechanical response of decompression alone in lower grade lumbar degenerative spondylolisthesis–A finite element analysis J Orthop Surg Res 2024 19 01 209 10.1186/s13018-024-04681-4 38561837 PMC10983632

[7] Yong-Hing K Kirkaldy-Willis W H The pathophysiology of degenerative disease of the lumbar spine Orthop Clin North Am 1983 14 03 491 504 6346204

[8] Panjabi M M The stabilizing system of the spine. Part I. Function, dysfunction, adaptation, and enhancement J Spinal Disord 1992 5 04 383 389, discussion 397 10.1097/00002517-199212000-00001 1490034

[9] Van Grafhorst J MP Peul W C Vleggeert-Lankamp C LA Decompression without Fusion in Patients with Low-Grade Degenerative Spondylolisthesis and Stenosis: Long-Term Patient-Reported Outcome World Neurosurg 2025 193 893 902 10.1016/j.wneu.2024.10.123 39491616

[10] Abdel-Fattah A R Bell F Boden L To fuse or not to fuse: The elderly patient with lumbar stenosis and low-grade spondylolisthesis. Systematic review and meta-analysis of randomised controlled trials Surgeon 2023 21 01 e23 e31 10.1016/j.surge.2022.02.008 35305933

[11] Karlsson T Försth P Öhagen P Michaëlsson K Sandén B Decompression alone or decompression with fusion for lumbar spinal stenosis: five-year clinical results from a randomized clinical trial Bone Joint J 2024 106-B 07 705 712 10.1302/0301-620X.106B7 38945544

[12] Kgomotso E L Hellum C Fagerland M W Decompression alone or with fusion for degenerative lumbar spondylolisthesis (Nordsten-DS): five year follow-up of a randomised, multicentre, non-inferiority trial BMJ 2024 386 e079771 39111800 10.1136/bmj-2024-079771 PMC11304163

[13] Unterfrauner I Laguna J M Serra-Burriel M Fusion versus decompression alone for lumbar degenerative spondylolisthesis and spinal stenosis: a target trial emulation with index trial benchmarking Eur Spine J 2024 33 11 4281 4291 10.1007/s00586-024-08495-0 39305301

[14] Cheng H Luo G Xu D Decompression alone or fusion in single-level lumbar spinal stenosis with spondylolisthesis? A systematic review and meta analysis BMC Musculoskelet Disord 2024 25 01 726 10.1186/s12891-024-07641-5 39256670 PMC11386329

[15] Khashan M Salame K Ofir D Lidar Z Regev G J Stable Low-Grade Degenerative Spondylolisthesis Does Not Compromise Clinical Outcome of Minimally Invasive Tubular Decompression in Patients with Spinal Stenosis Medicina (Kaunas) 2021 57 11 1270 10.3390/medicina57111270 34833488 PMC8622409

[16] Ghiselli G Wang J C Bhatia N N Hsu W K Dawson E G Adjacent segment degeneration in the lumbar spine J Bone Joint Surg Am 2004 86 07 1497 1503 10.2106/00004623-200407000-00020 15252099

[17] Carreon L Y Puno R M Dimar J R 2nd Glassman S D Johnson J R Perioperative complications of posterior lumbar decompression and arthrodesis in older adults J Bone Joint Surg Am 2003 85 11 2089 2092 10.2106/00004623-200311000-00004 14630835

